# Inconclusive results from an epidemiological study on dietary acrylamide and cancer

**DOI:** 10.1038/sj.bjc.6601016

**Published:** 2003-08-12

**Authors:** L Hagmar, M Törnqvist

**Affiliations:** 1 Department of Occupational and Environmental Medicine, Lund University Hospital, SE-221 85 Lund, Sweden; 2 Department of Environmental Chemistry, Stockholm University, SE-106 91 Stockholm

**Sir**,

The association between dietary acrylamide and cancer of the large bowel, kidney and bladder was assessed in a Swedish case–control study, with a total of 987 cases and 538 healthy controls, that originally had been created for evaluating the carcinogenic effects of heterocyclic amines from diet (Mucci *et al*, 2003). Individual dietary acrylamide intakes were crudely estimated from food frequency questionnaires and reported levels of acrylamide in frequently consumed food products. No associations were observed between dietary acrylamide and any cancer risk. The authors state: ‘The first study of dietary acrylamide in relation to three major human cancers is reassuring.’ We think that this is too strong a conclusion.

A basal aspect to consider when interpreting the Swedish results is the size both of the expected cancer risk enhancement and of the cancer risk possible to detect. The authors correctly state, ‘…a true association may be concealed if the level of exposure in the studied population is low and/or if the range of variation is limited.’ Unfortunately, the authors provide only limited information about the estimated daily intake of acrylamide among cases and controls, which makes it virtually impossible to get the full picture of absolute levels and interindividual variations in exposure. The interquartile comparisons given in cannot be interpreted in terms of absolute levels and variation of exposure. The only acrylamide dose figures given in the paper concern the estimated mean daily intakes (27.5 *μ*g among the controls and 28.4–29.4 *μ*g among the cases), and that less than 2% of the population was estimated to have a daily intake as high as 1 *μ*g acrylamide per kilogram body weight per day. If we assume an average body weight of 70 kg in the population, those 2% of the population with a ‘high’ exposure had an estimated excess intake of about 42.5 *μ*g acrylamide per day; this corresponds to an increase in lifetime cancer mortality of 2.7 per 1000, based on the risk assessment model of the United States Environmental Protection Agency (1990). An estimate based on a multiplicative model (Granath *et al*, 1999) would arrive at roughly nine extra cancer deaths per 1000 (Törnqvist *et al*, 1998). Assuming a cumulative total cancer mortality of about 18%, and also assuming that the carcinogenicity of acrylamide is not different with respect to fatal or nonfatal malignant neoplasms, relative risks for cancers of approximately 1.015 and 1.05, respectively, could be expected for those 2% of the Swedish study population with the highest daily intake of acrylamide as compared to the mean intake. It can be calculated that from a purely statistical point of view about 470000 (!) cases, with half as many controls, are needed in order to show a relative risk of 1.05 in a statistically significant way (*P*<0.05), and with 80% statistical power, among those 2% with ‘high’ exposure. It can also be calculated that, assuming an exposure prevalence of 2%, the lowest possible relative risk that would have 80% chance of being significantly detected in a study of 987 cases and 538 controls is as high as 2.4; this type of power calculation assumes that there is no residual confounding or misclassification bias. However, the Swedish study is likely to suffer from considerable nondifferential misclassification of exposure to acrylamide, taking into account both lack of precision in food frequency questionnaire data and the sparse data on acrylamide levels in food products that were available for the intake calculations. Thus, it is not realistic that not even a ‘true’ relative risk as high as 2.4 could have been detected in the Swedish study.

There is a need to validate the type of exposure assessments for acrylamide that were used in the Swedish study using biomarkers for acrylamide exposure, for example, haemoglobin adducts of acrylamide, which reflect the cumulative dose during the preceding months (Granath *et al*, 1992; Törnqvist *et al*, 2002). It needs to be assessed first, whether food frequency data can be used for quantifying dietary intake of acrylamide and second, the variation in acrylamide intake in the population. More specifically, we need to know whether there are sufficient subjects with high intake, as this is a prerequisite for the design of epidemiological studies with a prospect of evaluating the carcinogenicity to humans of dietary acrylamide.
